# Immunotherapy of Ovarian Cancer with Particular Emphasis on the PD-1/PDL-1 as Target Points

**DOI:** 10.3390/cancers13236063

**Published:** 2021-12-01

**Authors:** Janina Świderska, Mateusz Kozłowski, Sebastian Kwiatkowski, Aneta Cymbaluk-Płoska

**Affiliations:** 1 Department of Gynecological Surgery and Gynecological Oncology of Adults and Adolescents, Pomeranian Medical University in Szczecin, al. Powstańców Wielkopolskich 72, 70-111 Szczecin, Poland; jasia.swiderska@gmail.com (J.Ś.); aneta.cymbaluk@gmail.com (A.C.-P.); 2 Department of Obstetrics and Gynecology, Pomeranian Medical University in Szczecin, al. Powstańców Wielkopolskich 72, 70-111 Szczecin, Poland; kwiatkowskiseba@gmail.com

**Keywords:** immunotherapy, immune checkpoint, checkpoint inhibitor, ovarian cancer, PDL-1, PD-1

## Abstract

**Simple Summary:**

Ovarian cancer has remained the leading cause of death among gynecologic malignancies. The current standard of treatment, in most cases, is a combination of surgery and chemotherapy, based on platinum agents and taxanes. Despite the increasing usage of newer drug groups, such as bevacizumab and PARP inhibitors, and the expansion of patient groups for these drugs, ovarian cancer is characterized by recurrences, particularly in the form of peritoneal implants. This review focuses on immunotherapy for ovarian cancer. It considers the current state of knowledge in areas such as cancer vaccines, adoptive cell therapy, CAR-T therapy, and anti-CTLA-4 monotherapy. The paper specifically considers PD-1/PDL-1 as drug targets. Anti-PD-1/PD-L1 monotherapy, and anti-PD-1/PD-L1 immunotherapy in combination with other agents, are analyzed.

**Abstract:**

Ovarian cancer is one of the most fatal cancers in women worldwide. Cytoreductive surgery combined with platinum-based chemotherapy has been the current first-line treatment standard. Nevertheless, ovarian cancer appears to have a high recurrence rate and mortality. Immunological processes play a significant role in tumorigenesis. The production of ligands for checkpoint receptors can be a very effective, and undesirable, immunosuppressive mechanism for cancers. The CTLA-4 protein, as well as the PD-1 receptor and its PD-L1 ligand, are among the better-known components of the control points. The aim of this paper was to review current research on immunotherapy in the treatment of ovarian cancer. The authors specifically considered immune checkpoints molecules such as PD-1/PDL-1 as targets for immunotherapy. We found that immune checkpoint-inhibitor therapy does not have an improved prognosis in ovarian cancer; although early trials showed that a combination of anti-PD-1/PD-L1 therapy with targeted therapy might have the potential to improve responses and outcomes in selected patients. However, we must wait for the final results of the trials. It seems important to identify a group of patients who could benefit significantly from treatment with immune checkpoints inhibitors. However, despite numerous trials, ICIs have not become part of routine clinical practice for the treatment of ovarian cancer.

## 1. Introduction

Ovarian cancer is the fifth most prevalent and the fourth most fatal cancer in women globally [1,2]. About 239,000 new cases and about 152,000 deaths are reported worldwide each year [3]. Ovarian cancer has a high mortality to morbidity ratio and is the most common cause of death from gynecological tumors [1,2]. Due to the lack of early, specific symptoms, only about 15% of cases are diagnosed as early, stage I tumors [3]. In the case of advanced disease, the 5-year survival rate is as low as 29.2% [4]. The current first-line treatment standard is cytoreductive surgery combined with platinum-based chemotherapy [5]. However, attention should be paid to a relatively new group of drugs: Poly(ADP-ribose) polymerase (PARP) inhibitors, which have profoundly changed the prognosis of patients with ovarian cancer. Bevacizumab is another drug used for patients with ovarian cancer. Despite the use of novel drugs and widening the target groups of patients, ovarian cancer is characterized by recurrences. Therefore, there is an emerging need for a new therapeutic approach that will revolutionize the treatment of ovarian cancer. The introduction of innovative drugs based on immunological target points could be a hope, not only for treatment of cancer recurrences and prolongation of progression-free survival, but also in attempts to cure patients completely.

Abnormal cells are formed in every system during their lifetime. They are instantly recognized and eliminated by the immune system. This immune response against the mutated cells can be divided into acquired, i.e., targeted against specific antigens, and non-specific innate immune response [6]. The effectors of the innate response recognize and destroy tumor cells that have lost the MHC-I proteins. Elimination of MHC-I molecules is one of the mechanisms used by tumors to escape a specific response of the immune system [6,7]. The interaction between the immune system and cancer cells is greatly influenced by the tumor microenvironment, which includes numerous types of cells that release various types of chemokines, interleukins, and growth factors; thus increasing the proliferation, migration, and invasiveness of cancer cells; reducing the bioavailability of medicines; and causing local suppression of the immune system within the tumor [8].

The significant role of the immune system in tumor formation and growth is demonstrated by the fact that cancers are much more frequent in patients with congenital and acquired immune deficiencies [9]. As shown by Lee et al., preoperative lymphocytopenia was a negative prognostic factor in patients with advanced-stage ovarian cancer [10]. The presence of tumor infiltrating lymphocytes in ovarian cancer tissue has proven to be an independent prognostic factor for recurrence and survival in ovarian cancer patients [11,12]. The effect of the immune response on the reduction of proliferative disease was also demonstrated by the spectacular spontaneous resolution of diffuse intraperitoneal ovarian cancer following septic peritonitis, which had been developed as a complication following a specimen being collected from the great omentum [13]. Preclinical studies using a murine models of ovarian cancer have shown that intraperitoneal administration of interleukin-33, which stimulates the allergic reaction of the immune system, leads to regression of cancer and extends the recurrence-free survival. This approach is considered by authors as an interesting new target for ovarian cancer patients, especially those with peritoneal carcinomatosis [14,15].

**Figure 1:**
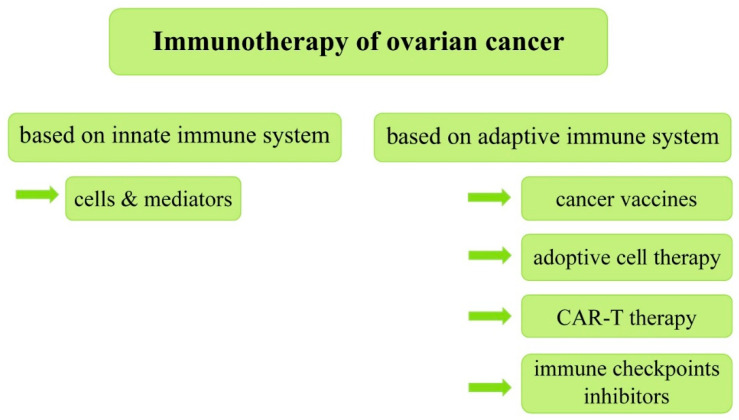
Classification of immunotherapy of ovarian cancer based on the innate and adaptive immune system.

Immunotherapies that have proven to be effective in a variety of other malignancies with a poor prognosis are the focal point of this article. The discovery of immune checkpoint inhibitors led to a breakthrough in malignant melanoma treatment, significantly improving survival rates in advanced metastatic non-operative melanoma from 20% (3-year survival) up to 53% (4-year survival) [16]. Checkpoint blockades are also registered for the treatment of various other cancers, such as advanced clear-cell renal carcinoma with intermediate and poor prognostic features, advanced metastatic non-small cell lung carcinoma, recurrent Hodgkin’s lymphoma, and recurrent hepatocellular carcinoma, following the failure of other treatments [17,18,19,20]. Regarding lung cancer, four anti-PD1/PD-L1 drugs (Nivolumab, Pembrolizumab, Atezolizumab, and Durvalumab) were approved by the FDA because of their significant impact on its treatment. Despite this, challenges remain in lung cancer immunotherapy, including identifying patients who may benefit from treatment, improving the therapeutic effect, and reducing immune-related adverse events. Studies show that based on anti-PD1/PD-L1 treatment combined with other therapies, such as radiotherapy, chemotherapy, targeted therapy, and other therapies, lung cancer treatment can be optimized and adverse events can be reduced [21]. Reports of the use of immune checkpoint blockades in the treatment of breast cancer also appear promising, illustrating the potential of harnessing the immune system to achieve clinical benefits in this disease. Studies have shown that PD-1/PD-L1 pathway antagonists can induce sustained clinical responses in some patients with metastatic triple negative breast cancer. It is essential to mention at this point that both the validation of these early results and attempts to extend immunotherapy to patients with HER-2+ and luminal breast cancer are ongoing. It should be added that there are clinical trials evaluating the inclusion of immunotherapy in adjuvant and neoadjuvant treatment [22]. The aim of this paper is to review current research on immunotherapy in the treatment of ovarian cancer (Figure 1). The authors have specifically considered immune checkpoints molecules, such as PD-1/PDL-1 as targets for immunotherapy.

## 2. Selected Immune Molecules as Components of the Immune Response against Cancer Cells

The idea of the immune system being deliberately used for cancer treatment dates back to the 1890s when a reduction in tumor size was observed after intratumoral bacteria injection [23]. Current immunotherapeutic methods include tumor cell antigen vaccines aimed at enhancing the ability of the body’s own lymphocytes to recognize and destroy cancer cells, as well as the administration of specific T cells targeting specific cancer antigens [24]. Unfortunately, these treatments have rarely proven effective.

### 2.1. Innate Immune Mechanisms and the Mechanisms of Resistance to Immunotherapy

The immune system is divided into the innate immune system and the adaptive immune system, and this division is based primarily on the response to infection. Innate immune mechanisms are nonspecific inflammatory processes. These mechanisms use innate immune cells, such as monocytes, macrophages, or natural killer cells. In addition, the mediators of the innate immune system are molecules produced by the organism, such as lipids and cytokines. Innate immune mechanisms can lead to tumor eradication, but can also lead to an immunosuppressive tumor microenvironment. Therefore, innate immune cells and/or the molecules produced by them are potentially druggable targets in cancers. Correct priming, activation, and recruitment of T lymphocytes in the tumor microenvironment is essential for the correct functioning of the adaptive immune response. Any factor that disrupts these processes contributes to the resistance to the immune checkpoint blockade. Mechanisms specifically involved in immune checkpoint blockade resistance include insufficient tumor antigenicity, tumor-intrinsic interferon-γ signaling, tumor-intrinsic loss of MHC, tumor dedifferentiation and stemness, and regulation by oncogenic signaling, with a particular focus on certain pathways (the WNT–β-catenin pathway, the mitogen-activated protein kinase (MAPK) pathway, the cyclin-dependent kinase 4 (CDK4)–CDK6 pathway) and the pathways induced by loss of PTEN [25]. Thus, some specific modifications of immune cells, cytokines, co-inhibitory receptors, and co-stimulatory receptors in the tumor microenvironment affect the antitumor immune response, resulting in resistance to immune checkpoint blockade. These factors can be produced by the immune cells of the tumor microenvironment or the cancer cells; by releasing substances such as TGF-beta, IL-10, or prostaglandin E2 into the extracellular matrix, as well as by membrane expression of molecules such as CTLA-4, PD-L1, or PD-L2, they create an immunosuppressive environment in the tumor [6,24].

### 2.2. Immune Checkpoints

Physiological checkpoints of the immune response are an essential link between suppressing the immune response following an infection and in preventing autoimmunity by promoting tolerance to body’s own tissues. However, production of ligands for checkpoint receptors may be a very powerful, and undesirable, immunosuppressive mechanism in neoplasms [23]. These mechanisms have not been fully understood and remain a field of ongoing research. Some of the better-characterized components of checkpoint mechanisms include the CTLA-4 protein and the PD-1 receptor with its PD-L1 ligand [6].

### 2.3. CTLA-4

CTLA-4 is a membrane protein that is present in activated lymphocytes. It is homologous to the CD28 protein responsible for the second step in the activation of T cells after the TCR receptor is bound by the antigen. Unlike CD28, CTLA-4 is responsible for suppressing the immune response. Both proteins bind the same ligands, B7-1 and B7-2, the difference consisting in the fact that the affinity CTLA-4 has for both ligands is 500–2500 times stronger [26].

### 2.4. PD-1/PD-L1

PD-1 is a transmembrane immunoglobulin-like protein. It is expressed in the thymus and, to a lesser extent, in the spleen. It is almost undetectable in peripheral leukocytes, except for activated T and B cells [27]. The role of this protein as a negative regulator of immune response was first demonstrated using PD-1−/− mice. The absence of PD-1 expression was shown to have caused a higher incidence of autoimmune diseases such as lupus-like proliferative glomerulonephritis, arthritis, and dilated cardiomyopathy [27,28,29]. The ligand for this receptor, PD-L1, was discovered in the following years. Administration of this ligand to activated T cells resulted in their proliferation being inhibited and the secretion of interferon gamma and interleukin 2 being reduced [30]. Under physiological conditions, PD-L1 is expressed on T and B cells, dendritic cells, macrophages, epithelial cells, myocardial cells, pancreatic islet cells, glial cells, keratinocytes, and the maternal–fetal barrier. PD-L1 expression is increased by interferon gamma [24,30]. Neoplastic cells make use of the PD-1/PD-L1 pathway by overexpressing the PD-L1 ligand on their surfaces, which results in suppression of the immune response targeting these cells.

## 3. Immunotherapy in Ovarian Cancer

### 3.1. Immunotherapy Based on Innate Immune Response

Soluble mediators such as lipids and cytokines can be used for innate immunity-stimulating treatment; alternatively, cell therapy may be used, e.g., using NK cells. Immune response mediators, such as interferons, have been shown to be cytostatic and cytotoxic to cancer cells, both in vitro and in vivo [31]. Recruitment of ovarian cancer patients is ongoing to a phase I trial of intraperitoneal macrophage therapy administered following previous stimulation with INF gamma-1b and peginterferon alpha-2b [7]. In previous reports examining cell lines from ovarian cancer and other malignancies, the study authors showed that when treated with interferons, monocytes differentiate towards M1 macrophages presenting with anti-tumor activity [32,33]. Clinical trials based on innate immune response are still in the early phase, and therefore, no drug has yet been approved.

### 3.2. Cancer Vaccines

There are various types of vaccines to activate the immune system against cancer cells, starting with whole cell tumor lysates, through to gene modified tumor vaccines, heat shock proteins, naked DNA, peptide-based vaccines, to dendritic cell vaccines [34]. Despite the promising results of the phase II study of the Vigil vaccine (DNA engineered autologous whole cell therapy, incorporating rhGMCSF and the bifunctional shRNA) as a maintenance after first line treatment, which showed prolonged RFS (recurrence free survival) (27 vs. 16 months) [35], a larger GOG (Gynecologic Oncology Group) study showed no difference in overall survival between patients receiving polyvalent vaccine-KLH conjugate + OPT-821 (an immunological adjuvant) versus OPT-821 alone [36]. Due to these results, the previously planned phase III trial (NCT00693342) was withdrawn. 

### 3.3. Adoptive Cell Therapy

Adoptive cell therapy comprises the transfer of diverse immune cells, including lymphocytes or NK cells with or without genetic modification, as well as TILs and CAR or TCR gene modified T cells or the transfer of other immune cells such as NK cells.

### Adoptive Cell Therapy Using Tumor Infiltrating Lymphocytes

**Figure 2:**
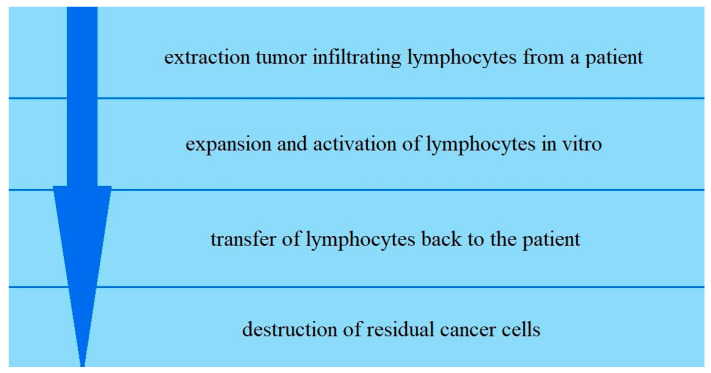
Scheme of the process of adoptive cell therapy.

Tumor infiltrating lymphocytes (TILs) are extracted from a patient’s tumor, expanded and activated in vitro, and then transferred back to the patient to destroy residual cancer cells [37] (Figure 2).

The success of this therapy depends crucially on the presence of tumor-specific lymphocytes that can recognize and destroy the tumor cells. The study of Freedman et al. showed no measurable responses when administering TILs intraperitoneally to patients with advanced refractory epithelial ovarian cancer [38]. In another study on a group of seven patients with refractory or advanced ovarian cancer treated with ACT as a monotherapy, one CR (complete response) and four PRs (partial response) were achieved. However, the regression of lesions was not durable, and lasted only 3–5 months [39]. Similarly, short effects and only stabilization of the disease was shown in another study evaluating the efficacy of ACT preceded by standard lymphodepleting chemotherapy in progressive, platinum resistant metastatic ovarian cancer. Interestingly, the authors emphasized the high frequency of expression of exhaustion markers, such as LAG3 and PD-1, on the surface of TILs obtained during the ACT procedure [40]. Adding the immune checkpoint inhibitor-anti CTLA-4 antibody to the TIL culture allows one to overcome such anergy by promoting the outgrowth of TILs and enhancing their anti-tumor reactivity [41]. Trials of adoptive cell therapy in combination with other checkpoint inhibitors are ongoing [42,43].

### 3.4. CAR-T Therapy

**Figure 3:**
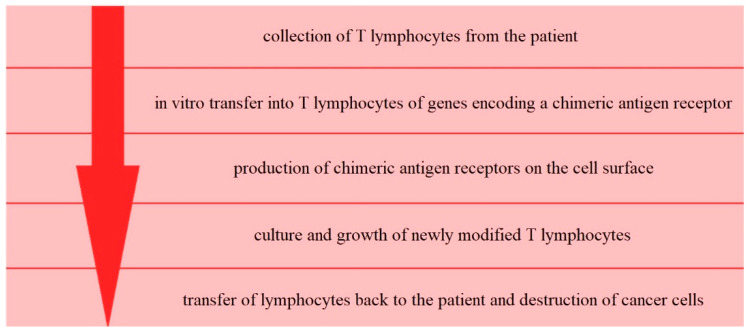
Scheme of the process of CAR-T therapy.

A more complex variant of adoptive cell therapy is chimeric antigen receptor-modified T cell therapy (CAR-T therapy) (Figure 3).

The idea behind this method is to genetically modify T cells collected from the patient, to increase their anti-tumor properties. To achieve this, genes coding a chimeric antigen receptor (recognizing specific tumor antigens) are transferred in vitro into T cells via viral or non-viral approaches [44]. The difficulty is that programming T cells to destroy cancer cells requires finding a specific feature of the mutant cell that strongly distinguishes it from other, healthy cells in the body. It is, therefore, essential to select the specific feature for the cancer cell because otherwise the T cells could target the healthy cells. Recognition of the target antigen by CAR-T cells leads to their activation, independently of the context of the MHC proteins. Thus, CAR-T cells can recognize antigens and destroy tumor cells without first recognizing antigens presented by MHC molecules. Current trials in ovarian cancer include CAR-T therapy targeting different tumor surface antigens such as mesothelin (NCT03814447, NCT03916679, NCT03799913, NCT03054298), folate receptor-α (NCT03585764), MUC16 (NCT03907527), TnMUC1 (NCT04025216), tyrosine protein kinase Met (NCT03638206), and CD70 (NCT02830724). There have also been two completed trials in ovarian cancer, with no results posted to date (NCT02541370, NCT02159716). CAR-T therapy is an interesting idea, with some significant drawbacks such as off-target side effects (due to lack of ideal target antigens expressed only in tumor cells), an immunosuppressive tumor microenvironment, unpredictable lymphocyte penetration into the solid tumor, high manufacturing costs, and treatment delays, due to the necessity of preforming ex-vivo transduction procedures for each patient individually [45,46].

### 3.5. Anti-CTLA-4 Monotherapy

Thus far there has only been one completed clinical trial of anti CTLA-4 monotherapy in ovarian cancer. It was an open label single arm trial NCT01611558 in which Ipilimumab was administered to patients with recurrent platinum-sensitive ovarian cancer. According to the results available on ClinicalTrials.gov (accessed on 8 May 2021), the best overall response rate was achieved in 10.3% patients and 42.5% of patients did not complete the induction phase of treatment due to drug toxicity [47].

### 3.6. Anti-PD-1/PD-L1 Monotherapy

The disruption of PD-L1 binding to its receptor is being widely examined in patients diagnosed with ovarian cancer. Clinical trials are ongoing on both anti-PD-1 and anti-PD-L1 antibodies. Most of the studies that have already been completed are phase I or phase II studies in patients with recurrent ovarian cancer and who had previously received several lines of treatment. A summary of the results of the completed anti-PD-1/PD-L1 monotherapy studies is presented in Table 1 [48,49,50]. Interestingly, response to the anti-PD-1/PD-L1 treatment was significantly more frequent in patients with less common, non-serous histopathological types of tumors [48,49,50,51]. In the KEYNOTE-28 study, the only patient presenting with CR had been histopathologically diagnosed with transitional cell carcinoma, while both patients presenting with PR had been diagnosed with adenocarcinoma [48]. In addition, in the Javelin solid tumor trial using avelumab, both patients with clear cell ovarian carcinoma met the irRECIST PR criteria, while two out of three patients with endometrial were able to present with CR. In the same study, CR was achieved by only seven out of 93 patients with serous ovarian carcinoma [51]. As it is known that better effects of ICB (immune checkpoint blockade) are seen in microsatellite instability cancer, as well as in those with a high mutational load (such as melanoma, or NSCLC (non-small-cell lung cancer) in smoker patients) [52], a higher response rate in clear cell ovarian cancer might be explained by a higher mutational load in this histological subtype. A highly immunogenic subgroup of clear cell ovarian carcinoma with microsatellite instability has also been documented, which might comprise potentially good responders to immunotherapy [49]. However, it is important to mention the resistance of various cancer cells to immune checkpoints blockade treatment. The mechanisms of resistance include interferon signaling, antigen presentation, WNT–β-catenin signaling, cell cycle regulatory signaling, mitogen-activated protein kinase signaling, and pathways activated by loss of the tumor suppressor phosphoinositide phosphatase PTEN [25]. Hence, molecular characterization of the tumor is of high importance, with particular emphasis on the molecular mechanisms of tumor-intrinsic resistance to immune checkpoint blockade. This would allow us to determine the respond of the patients to ICB treatment, which would expand the therapeutic tools available.

As shown by the data in Table 1, presenting the four trials focused on anti-PD-1/PD-L1 monotherapy, these therapies have a small proportion of patients who responded favorably to treatment (CR + PR: min. 8% and max. 15%). However, attention should be paid to the size of the study groups. Negative clinical data from trials confirm the lack of benefit for single agent PD-L1/PD1 inhibition in platinum-resistant ovarian cancer [53]. Despite the relatively low response to ICB monotherapy, the effect in patients who had achieved reemission or stable disease was frequently long-lasting. Thus, continuing the search for biomarkers of the response to ICB treatment is warranted [54]. 

### 3.7. Anti-PD-1/PD-L1 Immunotherapy in Combination with Other Agents

Due to the low response to monotherapy of immune checkpoint blockade agents in ovarian cancer patients, numerous attempts to use ICBs in combination with other agents are also being undertaken. 

Cancer cells subjected to chemotherapy are destroyed, releasing a large amount of cancer antigens and potentially exacerbating the immune response [6]. Combinations involving the PD-1/PD-L1 inhibitors include standard chemotherapeutics, PARP inhibitors, antiangiogenic drugs, anti-CTLA4 antibodies, and radiotherapy and are studied in neoadjuvant, adjuvant, maintenance, and recurrent disease treatment settings [55,56,57,58,59].

### 3.8. Double Checkpoint Inhibition

Recent studies examining immune cell lines have shown that PD-L1 can not only bind in trans with its receptor PD-1. The cis interaction with B7.1 (ligand for CTLA-4 and CD28) is also possible, when both are co-expressed on APCs. Such in-cis interaction on APCs disrupts PD-1/PD-L1 binding in trans, thus reducing the inhibitory effect of APCs on T lymphocytes. Thereby, antiPD-L1 monotherapy, by blocking not only PD-1/PD-L1 but also the PD-L1/B7.1 interaction may promote immunosuppressive action by binding B7.1 to CTLA-4 [60]. 

In a recently finished phase II trial comparing a combination treatment with nivolumab plus ipilimumab and nivolumab alone in ovarian cancer, the statistically significant advantage of combined treatment was shown. The response rate for the double checkpoint inhibition group was 31.4%, compared with 12.2% in the nivolumab alone group. Stable disease was achieved in 39% and 29% of patients, respectively. As in other ICB studies, patients with clear cell tumors appeared to benefit most. AEs (adverse events) occurred more often in the combined treatment group, albeit the difference was not statistically significant [61]. The better outcomes of patients treated with double checkpoint inhibition may be explained by the fact that both drugs act at different points of the immune response process. Anti-CTLA-4 plays a role during the early stages of an immune response [23], while anti-PD-1/PD-L1 antibodies act later on already activated T lymphocytes [30].

### 3.9. Anti-PD-1/PD-L1 Immunotherapy in Combination with Chemotherapy

Chemotherapy, not only destroys tumor cells by a cytotoxic or cytostatic mechanism, but is also known to stimulate anti-tumor immunity. The underlying mechanisms are the induction of immunogenic cell death and disturbing the immune-suppressive tumor microenvironment. Chemotherapy based on platinum agents downregulates immunosuppressive regulatory T cells, as well as increasing the tumor infiltration of CD8+ T cells and expression of major histocompatibility complex class I. Thus, a combination of platinum based chemotherapy with immune checkpoint inhibitors may be a booster of T cells effector function, which has already been demonstrated in lung cancer [62]. In July this year, the results of the first completed phase 3 clinical trial comparing immunotherapy (Avelumab) in combination with chemotherapy (pegylated liposomal doxorubicin) versus immuno- or chemotherapy alone in ovarian cancer were published. There were no statistically significant differences in PFS or OS between the groups. In a subgroup analysis, patients with PD-L1 and CD8 expression on tumor cells, and those without primary platinum resistance, tended to gain more from combination therapy. Unfortunately, this also did not reach statistical significance However, this draws attention to the need for a better selection of patients for further research [63]. The NINJA trial compared nivolumab versus gemcitabine (GEM) or pegylated liposomal doxorubicin (PLD) treatment for patients with platinum-resistant (advanced or recurrent) ovarian cancer. Histological types of ovarian cancer were considered in this trial. The most numerous group of non-serous carcinomas was clear cell carcinoma (*n* = 67). There was a demonstrated benefit of nivolumab in patients with clear cell ovarian cancer [64]. In turn, the PEACOCC trial investigated whether patients with advanced clear-cell ovarian cancer might benefit from treatment with pembrolizumab. There are several ongoing trials using such a combination of drugs.

### 3.10. Anti-PD-1/PD-L1 Immunotherapy in Combination with PARP Inhibitors

The combination of ICBs and PARP inhibitors (PARPis) appears promising. PARP is an enzyme responsible for the repair of DNA single-strand breaks. Its inhibition leads to the accumulation of neoantigens, which can be detected by the immune system. Furthermore, a less efficient DNA repair system results in accumulation of cytosolic DNA, which in turn causes an increased reaction of the immune system against cancer cells by release of interferons and chemoatractants in the pro-inflammatory cGAS-STING pathway. This, in turn, promotes the tumor microenvironment to enhance its anti-tumor response. Unfortunately, the proinflammatory effect of PARPis monotherapy is suppressed by the immune checkpoints, whose expression intensifies under the influence of interferons [65]. As expected, a synergistic effect of PARPis combined with anti-PD-1 was demonstrated in a preclinical trial in a murine ovarian cancer model [66]. Moreover, in a phase II trial in ovarian cancer patients carrying germ-line mutations of BRCA1 or BRCA2 genes, a synergistic effect of olaparib (PARPis), when combined with durvalumab (ICB), was demonstrated. The effect of the treatment was higher than that of PARP inhibitor monotherapy [44]. A summary of the results of the completed studies is presented in Table 2 [51,67,68]. When comparing Anti-PD-1/PD-L1 immunotherapy in monotherapy and with other agents, differences can be seen in regard to ORR. In the trials analyzed, the lowest ORR (CR + PR) was achieved by 14% of patients (durvalumab and olaparib were used) and the highest ORR was achieved by 71.9% of patients (durvalumab and olaparib were also used, but in a different treatment regimen). Differences in the response rates are mainly explained by the different treatment populations. Moreover, a combination of PARPi and PD-1 inhibitors with bevacizumab could even increase response rates, as shown in the MEDIOLA trial. Thus, the combination of anti-PD-1/PD-L1 agents with chemotherapy or PARPi can lead to increased response rates in selected patient populations. Nevertheless, as indicated by the data presented and also reported by Leary et al., ovarian cancer has not proven to be an ideal candidate for immune checkpoint inhibitors [53].

The ongoing phase III clinical studies are designed to verify the efficacy of immune checkpoint blockades in various configurations with standard chemotherapy, PARP inhibitors, and bevacizumab, in both the first and subsequent lines of treatment. These studies are summarized in Table 3 [56,71,72,73,74,75,76,77].

## 4. Conclusions

Thus far, the standard treatment for advanced ovarian cancer, consisting of surgery followed by chemotherapy, has not been efficient enough. A new approach to overcome patients’ poor prognosis is greatly needed. Immunotherapy, especially immune checkpoint inhibition, revolutionized treatment in other malignancies with low survival rates, and there were possibilities for a breakthrough in ovarian cancer treatment. However, this did not occur, for various suspected reasons, as well as some unknown.

To start with, in the clinical trials completed so far, PD-1/PD-L1 inhibitors were used in patients with recurrent ovarian cancer after several previous treatment lines. Meanwhile, Drakes and coworkers showed a significantly more frequent and higher PD-1 expression on the lymphocyte surface and PD-L1 on cancer cells in patients with early ovarian cancer stage than in those with advanced stage [23]. Clinically, better results were found when using Avelumab in patients, after no more than one prior chemotherapy line, compared to the total patients; 9.6% vs. 21% [51]. Next-generation/high throughput sequencing studies on resistant/refractory recurrent ovarian cancer tissue samples showed loss of hypermethylation I promotor region of BRCA gene and also reversions in germline BRCA1/2 mutation, which may led to higher genome stability [78]. As tumors with high mutational load are believed to be more immunogenic and respond better to immunotherapy, this may hold promise for better responses to immunotherapy in first-line treatment. In order to confirm or exclude this thesis, one has to wait for the results of ongoing clinical trials with the use of checkpoint inhibitors in patients undergoing first-line treatment.

Second, there is the lack of appropriate selection of patients before ICB treatment. As a group of long-lasting responders was seen in completed trials, the search for biomarkers is ongoing. In one study, a positive correlation between PD-L1 expression in tumor cells and response to nivolumab treatment was noted. However, there is high heterogeneity in PD-L1 expression between different samples of the same tumor, as has already been demonstrated for squamous cell carcinoma of the head and neck, breast, and gastric cancers. In cases of recurrent disease, often, only specimens from the first cytoreductive surgeries are available, which might misrepresent the current immunologic status of the patient [52]. 

Finally PD-1/PD-L1 and PD-L1/B7.1 cis interactions may also cause confusion. Co-expression of both PD-L1 and PD-1 and their interaction in cis on tumor cells inhibits the ability of PD-L1 in trans binding. Depending on the level of expression of those ligand-receptor pairs in different cells in patient, it is possible that by inhibiting anti-PD-1 we can promote PD-1/PD-L1 in trans immunosuppressive signaling [79]. 

In conclusion, immune checkpoints inhibitor therapy does not have a favorable prognosis in ovarian cancer patients, although the combination of anti-PD-1/PD-L1 agents with chemotherapy or PARPi can lead to increased response rates in selected patient populations. First-line polytherapy combining chemo-therapy, anti-angiogenic antibodies, and immunotherapy appears to be the most promising. However, we must wait for the final results of the trials. It is also important to consider whether the benefits of polytherapy outweigh the potential complications of this kind of treatment. It should be noted that immunotherapy may be most effective for ovarian cancers other than the high-grade serous subtype. It seems essential to identify a group of patients who could benefit significantly from treatment with immune checkpoints inhibitors.

## Figures and Tables

**Table 1:** Phase I or phase II studies in patients with recurrent ovarian cancer.

Study Title	Study Drug	Study Type	No. of Patients	Previous Chemotherapy Lines	Treatment Response						
					CR	PR	SD	CR +PR	Disease Control CR + PR +SD	Median PFS Months	Median OS Months
KEYNOTE-28	Pembrolysumab (Humanized IgG4) 10 mg/kg i.v. every 3 weeks	Phase 1b Open label	26	0–>5	3.8%	7.7%	26.9%	11.5%	38.4%	1.9	13.8
KEYNOTE-100	Pembrolysumab (Humanized IgG4) 200 mg i.v. every 3 weeks	Phase II study Open label	376	1–6	1.9%	6.1%	29.3%	8%	37%	2.1	Not achieved
UMIN000005714	Nivolumab (Human IgG4) 1 mg/kg or 3 mg/kg i.v. every 2 weeks	Phase II study Open-label	20	>2	10%	5%	30%	15%	45%	3.5	20
JAVELIN Solid Tumor Trial	Avelumab (Fully Humanized IgG1) 10 mg/kg i.v. every 2 weeks.	Phase 1b Open label	125	n.a.	0.8%	8.8% (12% according to the modified irRECIST criteria)	42.2%	9.6%	51.8%	2.6	11.2

CR—complete response, PR—partial response, SD—stable disease, PFS—progression-free survival, OS—overall survival, n.a.—not applicable.

**Table 2:** Completed clinical studies including polytherapy in patients with ovarian cancer.

Trial	CI + PARPi	N	Population	ORR (%)	DCR (%)	Reference
NCT02484404	Durvalumab + Olaparib	35	Platinum resistant 83% gBRCAm; 17% BRCAwt; 83%	14	37	Lee et al., ESMO 2018 [69]
MEDIOLA	Durvalumab + Olaparib	34	gBRCAm, platinum sensitive	71.9	80 at 12 weeks	Drew et al., ESMO 2019 [67]
	Durvalumab + Olaparib+ Bevacizumab	32	gBRCA WT platinum sensitive	34.4	28.1 at 24 weeks	Drew et al. ESMO 2020 [70]
		31	gBRCA WT platinum sensitive	87.1	77.4 at 24 weeks	
TOPACIO/Keynote-162	Pembrolizumab + Niraparib	62	Platinum –resistant or platinum unable tBRCA wild type 79%	18	65	Konstantinopoulos et al., Jama Oncol 2019 [68]

ORR—objective response rate, DCR—disease control rate.

**Table 3:** Phase 3 clinical trials in patients with ovarian cancer.

Study Title or Number	Enrolled Patients	Study Treatment	Study Endpoints	Comments
JAVELIN OVARIAN PARP 100	Patients with locally advanced or metastatic ovarian cancer Stage III or IV.	- chemotherapy + avelumab (Anti-PD-L1) followed by maintenance treatment with avelumab (Anti-PD-L1)and thalasoparib - chemotherapy followed by maintenance treatment with thalasoparib - chemotherapy + bevacizumab followed by maintenance treatment with bevacizumab	PFS	Enrollment stopped after JAVELIN Ovarian 100 study results were presented; the expected efficacy of avelumab in the first-line treatment of non-preselected patient population was not achieved.
DUO-O	Newly diagnosed patients with ovarian cancer, primary peritoneal cancer, or fallopian tube cancer. Stage III or IV.	- platinum-based chemotherapy + bevacizumab followed by maintenance treatment with bevacizumab and olaparib. - platinum-based chemotherapy + bevacizumab + durvalumab (Humanized IgG1) followed by maintenance treatment with bevacizumab and durvalumab (Humanized IgG1). - platinum-based chemotherapy + bevacizumab + durvalumab (Humanized IgG1) followed by maintenance treatment with bevacizumab, durvalumab (Humanized IgG1), and olaparib. - in tBRCAm patients: platinum-based chemotherapy + bevacizumab + durvalumab (Humanized IgG1) followed by maintenance treatment with bevacizumab, durvalumab (Humanized IgG1), and olaparib.	Primary: PFS Secondary: OS, PFS2, HRQoL, pCR, PK, ORR, DoR, TFST, TSST, TDT	Randomized, quadruple-blinded, placebo-controlled. Ongoing recruitment. Estimated Primary Completion Date 06/2023.
ATHENA	Newly diagnosed patients with epithelial ovarian cancer, primary peritoneal cancer, or fallopian tube cancer who had achieved complete or partial response to the first line of chemotherapy	Maintenance treatment: - rucaparib + nivolumab (Human IgG4) - rucaparib - nivolumab (Human IgG4) - placebo	Primary: PFS Secondary: OS, ORR, DoR, AEs, treatment safety and tolerance	Randomized, quadruple-blinded, placebo-controlled. Completed target enrollment. Estimated primary completion date 12/2024.
NCT02839707	Patients with recurrent ovarian cancer/primary peritoneal cancer/fallopian tube cancer	- Pegylated lysosomal doxorubicin + atezolizumab (Humanized IgG1k) - Pegylated lysosomal doxorubicin + atezolizumab (Humanized IgG1k) + bevacizumab - Pegylated lysosomal doxorubicin + bevacizumab	Primary: DLT, PFS, OS Secondary: ORR, AE, PRO, PD-L1 expression	Phase II and phase III study. Open-label. Ongoing recruitment. Estimated Primary Completion date 06/2023.
IMagyn050	Patients with newly diagnosed ovarian cancer, fallopian tube cancer, or peritoneal cancer Stage III or IV. Following PDS R = 2 or following neoadjuvant chemotherapy and IDS	- Paclitaxel + carboplatin + atezolizumab (Humanized IgG1k) + bevacizumab, followed by maintenance therapy with atezolizumab (Humanized IgG1k)+ bevacizumab - Paclitaxel + carboplatin + bevacizumab, followed by maintenance therapy with bevacizumab	Primary: PFS, OS, including separate determination in PD-L1-positive patients. Secondary: OR, DoR, HRQoL, AEs, ADAs	Randomized, double-blinded, placebo-controlled trial. Enrollment complete. Estimated Primary Completion Date 12/2022.
ANITA	Patients with recurrent, platinum-sensitive ovarian cancer.	- Platinum-based chemotherapy with subsequent maintenance niraparib - Platinum-based chemotherapy + atezolizumab (Humanized IgG1k) with subsequent maintenance niraparib + atezolizumab (Humanized IgG1k)	Primary: PFS Secondary: OS, TFST, TSST, PFS2, AE, ORR, DoR, PROs, HRQoL, and the dependence of the above on the BRCA, PK, ATA status	Randomized, triple-blinded, placebo-controlled. Ongoing recruitment. Estimated Primary Completion Date 08/2024.
ATLANTE	Patients with platinum-sensitive recurrence of epithelial ovarian cancer, primary peritoneal cancer, or fallopian tube cancer.	- Platinum-based chemotherapy + Avastin - Platinum-based chemotherapy + Avastin + atezolizumab (Humanized IgG1k ) with subsequent maintenance atezolizumab (Humanized IgG1k)	Primary: PFS Secondary: OS, TSST, AEs	Randomized, triple-blinded. Enrollment complete. Estimated Primary Completion Date 10/2021.
NCT03353831	Patients with first or second recurrence of ovarian cancer, Fallopian tube cancer or primary peritoneal cancer within <6 months since the last treatment. Along with patients with third disease recurrence.	- Chemotherapy: paclitaxel or pegylated liposomal doxorubicin + bevacizumab - Chemotherapy: paclitaxel or pegylated liposomal doxorubicin + bevacizumab + atezolizumab (Humanized IgG1k)	Primary: OS, PFS Secondary: QLQ, ORR, DOR	Randomized, partially blinded. Ongoing recruitment. Estimated Primary Completion Date 12/2023.
MK-7339-001/KEYLYNK-001/ENGOT-ov43	Patients with newly diagnosed low-differentiated ovarian cancer, primary peritoneal cancer, and stage III or IV ovarian cancer after PDS or planned for IDS.	- Chemotherapy: carboplatin and paclitaxel + pembrolysumab (Humanized IgG4), followed by maintenance olaparib and pembrolysumab (Humanized IgG4) - Chemotherapy: carboplatin and paclitaxel + pembrolysumab (Humanized IgG4), followed by maintenance pembrolysumab (Humanized IgG4) - Chemotherapy: carboplatin and paclitaxel * addition of bevacizumab is permitted in both study arms.	Primary: OS, PFS Secondary: PFS2, AES, treatment discontinuation due to AEs, QoL, TFST, TSST, TDT, PCR, Twist,	Randomized, quadropoly blinded. Completed target enrollment. Estimated primary completion date 10/2023.

PFS—progression-free survival, OS—overall survival, PFS2—second progression, HRQoL—health-related quality of life, pCR—pathological complete response, PK—the pharmacokinetics, ORR—objective response rate, DoR—duration of response, TFST—time to first subsequent therapy, TSST—time to second subsequent therapy, TDT—time to discontinuation or death, AEs—adverse events, DLT—dose limiting toxicities, PRO—patient reported outcomes, ADAs—anti-drug antibodies, QLQ—Quality of Life Questionnaire, QoL—quality of life.
